# Interventional Therapy for Upper Extremity Phlegmasia Cerulea Dolens in a Patient With Heparin-Induced Thrombocytopenia: A Case Report

**DOI:** 10.7759/cureus.57987

**Published:** 2024-04-10

**Authors:** José Miguel Hidalgo Oviedo, Julián Andrés Muñoz Durán, Brayan Muñoz-Caicedo, Johan Sebastian Lopera Valle, Maribel Plaza Tenorio

**Affiliations:** 1 Department of Interventional Radiology, Hospital Pablo Tobón Uribe, Medellín, COL; 2 Department of Radiology, Universidad de Antioquia, Medellín, COL; 3 Department of Interventional Radiology, San Vicente Fundación, Medellín, COL; 4 Department of Vascular Medicine, Hospital Pablo Tobón Uribe, Medellín, COL

**Keywords:** fondaparinux, hit, heparin-induced thrombocytopenia, therapeutic thrombolysis, computed tomography, phlegmasia cerulea dolens, upper extremity deep vein thrombosis

## Abstract

This article presents a case of a multimorbid 63-year-old woman with chronic kidney disease and heparin-induced thrombocytopenia (HIT). Following the insertion of a central venous catheter, she developed phlegmasia cerulea dolens (PCD) in her left arm, a rare and severe complication of deep vein thrombosis (DVT). Given the severity of the case, adapting to anticoagulant contraindications or unavailability, management with catheter-directed thrombolysis and mechanical thrombectomy was made. It is concluded that catheter-directed thrombolysis and mechanical thrombectomy are valuable therapeutic alternatives in critical situations where treatment options are limited.

## Introduction

Upper extremity deep vein thrombosis (UEDVT) is defined by subclavian, axillary, or brachial vein clots. Its prevalence has increased, representing nearly 10% of deep vein rhrombosis (DVP) nowadays, and is likely related to the widespread use of intravascular devices, particularly those for peripheral insertion [1,2]. The most severe clinical manifestation of UEDVT is phlegmasia cerulea dolens (PCD), a rare condition characterized by extensive peripheral venous thrombosis resulting in edema, cyanosis, and severe limb pain. Although PCD is classically described in the lower extremities, by definition, it also can occur in the upper extremities [1,3]. PCD can be triggered by multiple factors, including local vascular injuries and systemic hypercoagulable states [4]. Early recognition and prompt treatment initiation are crucial due to the potential ischemia or amputation risks and mortality associated with PCD [5].

This case details a patient with end-stage renal failure with a central venous catheter who developed heparin-induced thrombocytopenia (HIT) and subsequently PCD. The aim is to elucidate the clinical progression, diagnostic findings, and the unique treatment challenges encountered, which include the implementation of thrombolysis necessitated by contraindications to fondaparinux and the unavailability of argatroban, in managing this severe complication arising from intravascular devices in a hypercoagulable state patient.

## Case presentation

A 63-year-old woman with chronic kidney disease, type 2 diabetes mellitus, peripheral artery disease, and hypertension consulted the emergency department of another hospital, complaining of exertional dyspnea, chest pain, and lower limb claudication persisting for three months alongside an inflamed ulcer on her left leg. She underwent surgical debridement of the ulcer and received antibiotic therapy. Arterial Doppler examination revealed significant stenosis in her lower extremities, prompting referral to a tertiary center. Despite recommendations for hemodialysis and endovascular intervention, she opted for conservative medical management and was subsequently discharged.

Three days post-discharge, she was readmitted with a hypertensive emergency, heart failure, and worsening chronic kidney disease. Hemodialysis was initiated via a left internal jugular vein catheter, which failed, leading to the placement of a new catheter in the right internal jugular vein. During this process, she developed severe thrombocytopenia, requiring platelet transfusion. She was later diagnosed with HIT.

**Figure 1:**
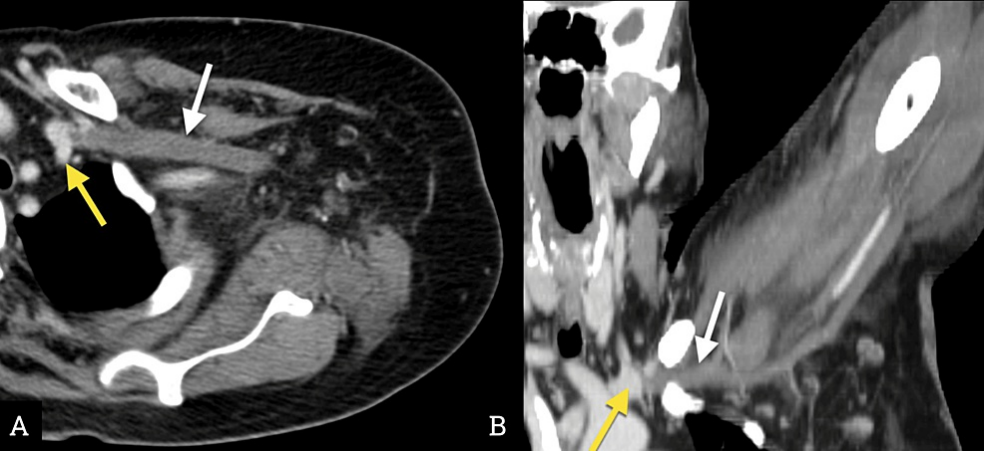
Venous phase angiotomography with axial (A) and coronal reconstructions (B) in maximum intensity pixels (MIP). A hypodense image occupies the lumen in the left subclavian vein (white arrows), consistent with thrombosis. The vessel permeability interface is demarcated (yellow arrows).

During her hospital stay, she developed severe pain and ischemic changes in her left leg, accompanied by persistent pain in her left arm. In critical condition, she was transferred to our hospital, where an infracondylar amputation of the left leg was performed due to severe ischemia. Additionally, she presented with pain and edema in her left arm. A CT angiography revealed extensive acute venous thrombosis from the subclavian vein to the humeral confluence on the left side (Figure 1).

**Figure 2:**
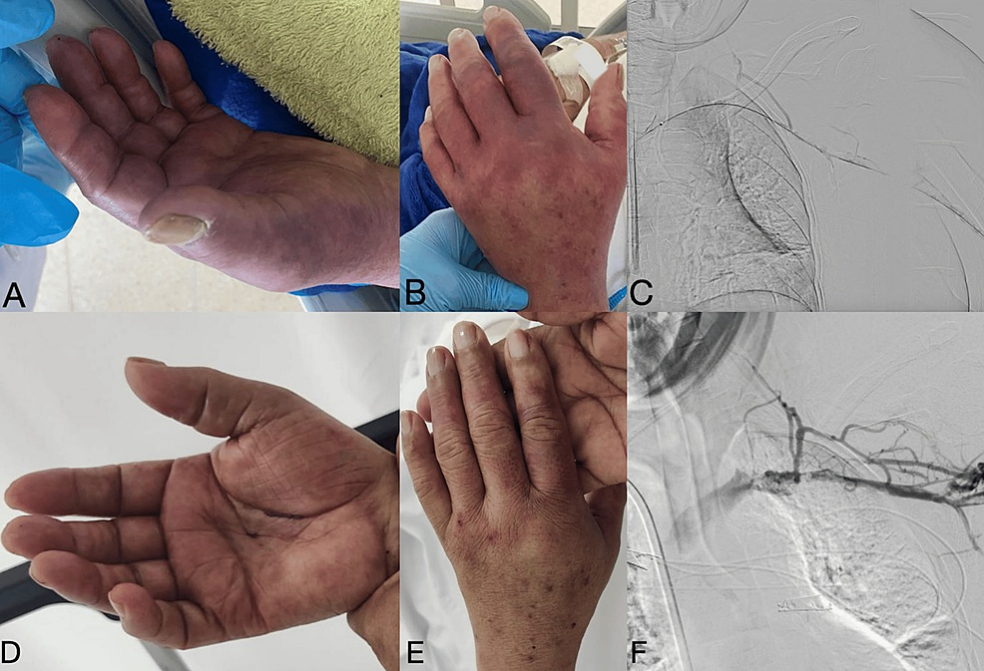
Photographs of the left upper limb and digital subtraction angiography. Before (A, B, and C) and 12 hours after (D, E, and F) treatment with catheter-directed thrombolysis, mechanical thrombectomy, and recombinant tissue plasminogen activator infusion. Photos with the patient’s consent.

The patient's condition progressed to PCD. Due to fondaparinux contraindication and the unavailability of argatroban, catheter-directed thrombolysis with recombinant tissue plasminogen activator (rTPA), and subsequent mechanical thrombectomy using AngioJet ZelanteDVT™ was performed. This intervention successfully restored perfusion to the upper limb (Figure 2). The patient was discharged after a prolonged hospitalization with controlled pathologies, anticoagulation, and ambulatory center access to hemodialysis.

## Discussion

Before the 1970s, UEDVT was a relatively rare entity, comprising less than 2% of all DVT cases. However, its prevalence has escalated to 10%, likely due to the increased use of central venous catheters and transvenous pacemakers [1,2,6]. Also, the hypercoagulable states apply to higher frequencies, requiring special mention of the association of HIT, a hypercoagulable acquired state, with central venous catheters, which increases the frequency of UEDVT (p = 0.00035) [4,5].

The reference standard for upper extremity thrombosis diagnosis is contrast venography. Compared with this technique, studies of computed tomography venography in the upper extremity have reported concordance of up to 100% [7]. For magnetic resonance venography, the data is variable, with concordance reported to be up to 100%, while other authors report sensitivity and specificity of 50% and 80%, respectively, in contrasted studies. For the non-contrasted sequences like time-of-flight looking for thrombosis, the sensitivity is between 71% and 97%, and the specificity is between 89% and 94%. For the ultrasound, the preferred and initial method of investigation when UEDVP is suspected, the sensitivity and specificity are around 97% and 96%, respectively [2].

PCD represents the most severe form of DVT that occurs around 2%-5% of all UEDVT, which endangers the limb and demands prompt recognition and early intervention. It is characterized by venous outflow tract obstruction, resulting in edema, cyanosis, and refractory pain in the affected limb. If left untreated, this condition may escalate to increased interstitial pressure, eventually leading to arterial collapse with a reduction in capillary flow, ischemia, and rapid progression to venous gangrene, risking limb loss and death [1,3].

A systematic review of 38 patients with PCD revealed that the commonest comorbidities associated were malignancy (37%), congestive heart failure (26%), and infection (26%). Also, the mortality to 60 days was 61%, with the amputation risk of 34% [1].

The optimal treatment strategy for PCD has yet to be definitively established. Immediate anticoagulation therapy and extremity elevation should constitute the initial approach. Therapeutic options such as systemic thrombolysis, surgical thrombectomy, and catheter-directed thrombolysis are available for patients with severe venous outflow obstruction and impending risk of progressing to venous gangrene [1,3].

Studies indicate that catheter-directed thrombolysis is associated with a substantial reduction in the risk of post-thrombotic syndrome and improves the likelihood of limb preservation [6,8-10]. Conversely, some case reports have posited that intravenous thrombolysis or mechanical thrombectomy may not be effective in salvaging the limb in advanced stages, such as venous gangrene, with thrombolysis success rates around 60% [11,12]. For cases of PCD complicated with compartment syndrome, surgical fasciotomy is indicated [1]. Then, given the management possibilities and variable results, the evidence of these scarce cases needs to be published to increase the data on intervention results and establish the optimal standard of care.

Finally, our patient had many risk factors for PCD, like heart failure, upper left jugular vein recent vascular access, upper right jugular vein catheter, infection, and a hypercoagulable state. The contraindication to anticoagulants or their unavailability limited the therapeutic options. So, endovascular therapy successfully improved venous outflow with remarkable clinical improvement and no amputation of the affected limb to discharge. However, we recognize the inherent limitations of this case report, and its results are not generalizable. For this reason, we encourage conducting more studies to optimize the management of PCD in the upper extremities.

## Conclusions

PCD in the upper extremities has high morbidity and mortality rates, with rapid progression representing a clinical challenge. We must suspect it in cases of extensive upper extremity venous thrombosis for timely diagnosis and treatment. This case also demonstrates the complex interaction between localized vascular injury from a central venous catheter and a systemic hypercoagulable disorder, including HIT, which led to UEDVT complicating with PCD. In such acute settings, catheter-directed thrombolysis and mechanical thrombectomy have emerged as viable therapeutic options, particularly when conventional treatments are contraindicated or unavailable. More studies are needed to improve management options.
